# Hidden in Plants—A Review of the Anticancer Potential of the Solanaceae Family in In Vitro and In Vivo Studies

**DOI:** 10.3390/cancers14061455

**Published:** 2022-03-11

**Authors:** Tomasz Kowalczyk, Anna Merecz-Sadowska, Patricia Rijo, Mattia Mori, Sophia Hatziantoniou, Karol Górski, Janusz Szemraj, Janusz Piekarski, Tomasz Śliwiński, Michał Bijak, Przemysław Sitarek

**Affiliations:** 1 Department of Molecular Biotechnology and Genetics, University of Lodz, 90-237 Lodz, Poland; tomasz.kowalczyk@biol.uni.lodz.pl; 2 Department of Computer Science in Economics, University of Lodz, 90-214 Lodz, Poland; anna.merecz-sadowska@uni.lodz.pl; 3 CBIOS—Research Center for Biosciences & Health Technologies, Universidade Lusófona de Humanidades e Tecnologias, 1749-024 Lisbon, Portugal; p1609@ulusofona.pt; 4 iMed.ULisboa—Research Institute for Medicines, Faculdade de Farmácia da Universidade de Lisboa, Av. Prof. Gama Pinto, 1649-003 Lisbon, Portugal; 5 Department of Biotechnology, Chemistry and Pharmacy, University of Siena, 53100 Siena, Italy; mattia.mori@unisi.it; 6 Laboratory of Pharmaceutical Technology, Department of Pharmacy, School of Health Sciences, University of Patras, 26504 Patras, Greece; sohatzi@upatras.gr; 7 Department of Clinical Pharmacology, Medical University of Lodz, 90-151 Lodz, Poland; karol.gorski@umed.lodz.pl; 8 Department of Medical Biochemistry, Medical University of Lodz, 92-215 Lodz, Poland; janusz.szemraj@umed.lodz.pl; 9 Department of Surgical Oncology, Chair of Oncology, Medical University in Lodz, Nicolaus Copernicus Multidisciplinary Centre for Oncology and Traumatology, 93-513 Lodz, Poland; janusz.piekarski@umed.lodz.pl; 10 Laboratory of Medical Genetics, Faculty of Biology and Environmental Protection, University of Lodz, 90-236 Lodz, Poland; tomasz.sliwinski@biol.uni.lodz.pl; 11 Biohazard Prevention Centre, Faculty of Biology and Environmental Protection, University of Lodz, 90-236 Lodz, Poland; michal.bijak@biol.uni.lodz.pl; 12 Department of Biology and Pharmaceutical Botany, Medical University of Lodz, 90-151 Lodz, Poland

**Keywords:** anticancer potential, apoptosis, cytotoxic effect, in vitro and in vivo studies, plant extracts, pure compounds, Solanaceae

## Abstract

**Simple Summary:**

The Solanaceae family is one of the most important arable and economic families in the world. In addition, it includes a wide range of valuable active secondary metabolites of species with biological and medical properties. This literature review focuses on the assessment of the anticancer properties of the extracts and pure compounds, and the synergistic effects with chemotherapeutic agents and nanoparticles from various species of the Solanaceae family, as well as their potential molecular mechanisms of action in in vitro and in vivo studies in various types of tumours.

**Abstract:**

Many of the anticancer agents that are currently in use demonstrate severe side effects and encounter increasing resistance from the target cancer cells. Thus, despite significant advances in cancer therapy in recent decades, there is still a need to discover and develop new, alternative anticancer agents. The plant kingdom contains a range of phytochemicals that play important roles in the prevention and treatment of many diseases. The Solanaceae family is widely used in the treatment of various diseases, including cancer, due to its bioactive ingredient content. The purpose of this literature review is to highlight the antitumour activity of Solanaceae extracts—single isolated compounds and nanoparticles with extracts—and their synergistic effect with chemotherapeutic agents in various in vitro and in vivo cancer models. In addition, the biological properties of many plants of the Solanaceae family have not yet been investigated, which represents a challenge and an opportunity for future anticancer therapy.

## 1. Introduction

Cancer is arguably one of the most dangerous diseases for civilization, affecting all people, regardless the their origin, age or social status. Cancer is also one of the most common causes of death after cardiovascular diseases in developed countries. Statistics show that about 20 million new cases were diagnosed in the world in 2020, and as many as 10 million people died as a result of cancer [1]. Apart from the genetic determinants of the development of cancer (e.g., the presence of typical gene mutations), the most common causes of cell transformation are lifestyle factors (e.g., radiation exposure, smoking, poor diet, alcohol consumption, occupational factors or environmental contamination). Gender and age are also of great importance in the development of neoplastic diseases, and have a significant influence on cancer incidence and mortality [2,3,4,5]. Tumour development and progression are complex, involving factors in the cancer cells themselves like abnormally excessive proliferation as well as multidimensional interactions between other cells and tissues in the body. These cells can also cause angiogenesis, and are capable of dividing indefinitely and metastasizing. As such, rapid and correct diagnosis increases the chances of successful treatment [6,7].

Statistics show that one in six deaths in the world today is caused by cancer, and about 70% of these deaths occur in low- and middle-income people [8]. The problem of increased cancer incidence around the world has led to an increasing search for new compounds that are ideally toxic to cancer cells but not to normal cells. As the currently used chemotherapeutic agents show relatively high toxicity towards both neoplastic cells and normal cells [9,10,11], there is great interest in the identification of new compounds of natural origin with specific activity against different cancer types. The anticancer properties of plants have been recognised for centuries. About 60% of the drugs currently used to treat cancer were originally isolated from nature, with the plant kingdom being the most important source. The intensive development of phytochemistry and isolation methods of plant-derived compounds eventually led to the development of a number of anticancer drugs [12].

A considerable variety and number of plants are known to have medicinal properties [13,14,15]. An estimated 70,000 plant species, from algae to trees, have been used for medicinal purposes [16]. The National Cancer Institute (NCI) has studied approximately 35,000 plant species for potential anticancer effects. Among them, about 3000 plant species showed reproducible anticancer activity [8]. While the bioactive principles from these plants can be obtained traditionally by extraction from various natural sources, they can be produced more efficiently using various biotechnological tools. The range of secondary metabolites known to exhibit anticancer properties is chemically broad, with the predominant groups being alkaloids, terpenoids and polyphenols [17,18].

Both pure compounds and extracts of plant origin show cytotoxic effects by inducing apoptosis in cancer cells [19,20,21,22,23,24]. Secondary metabolites, either used per se or as nanoparticles in targeted therapy, have also been found to have numerous effects on cancer, both in vitro and in vivo; they also appear to interact with chemotherapeutic agents, thus positively or negatively affecting their efficacy, and to protect normal cells against the adverse effects of anticancer therapies [25,26,27].

One family of plants commonly included in the diet is the Solanaceae. It is one of the largest plant families, and its genus—*Solanum*—is the richest in edible species, including potatoes (*Solanum tuberosum*), tomatoes (*Solanum lycopersicum*) and eggplants (*Solanum melongena*); in addition, peppers are widely-consumed vegetables which are included in the related genus *Capsicum* [28,29]. The *Solanum* genus contains a range of phenolic, alkaloid, saponin, terpene and lipid compounds; as such, it has frequently been used for medicinal purposes. Many of the alkaloids from the *Solanum* genus are particularly interesting because they have demonstrated extensive antirheumatic, antimicrobial, antioxidant and antitumour effects, in the latter case against several types of cancer [30,31]. 

The present study displays selected general issues including medicinal plants in cancer treatment, nanotechnology and plant compounds in the fight against cancer, the synergy between chemotherapeutic agents and plant compounds in cancer therapy, and the Solanaceae family’s general characteristics and application. Moreover, the reports discussed the functional properties of extracts or compounds isolated from Solanaceae species that are known to exert selective anticancer activity in vitro and in vivo, as well as their synergistic effects when used in combination with chemotherapeutic agents. In some cases, the extracts were formulated by using nanoparticle-based delivery systems. The studies also address the molecular and cellular mechanisms involved in the death of cancer cells.

## 2. Inclusion and Exclusion Criteria

This research papers included in this review focused on in vitro and in vivo studies of plant extracts or isolated compounds from the Solanaceae family administered per se, or in combination with chemotherapeutics. In certain studies, the potential mechanisms of antitumour activity were discussed. Only research articles published in peer-reviewed journals were selected. Studies in which the isolated compounds of interest were synthesized or purchased, rather than being derived from plant material, were excluded. In addition, studies that did not specify the extraction and/or purification methodology of a bioactive compound were also excluded. 

**Figure 1:**
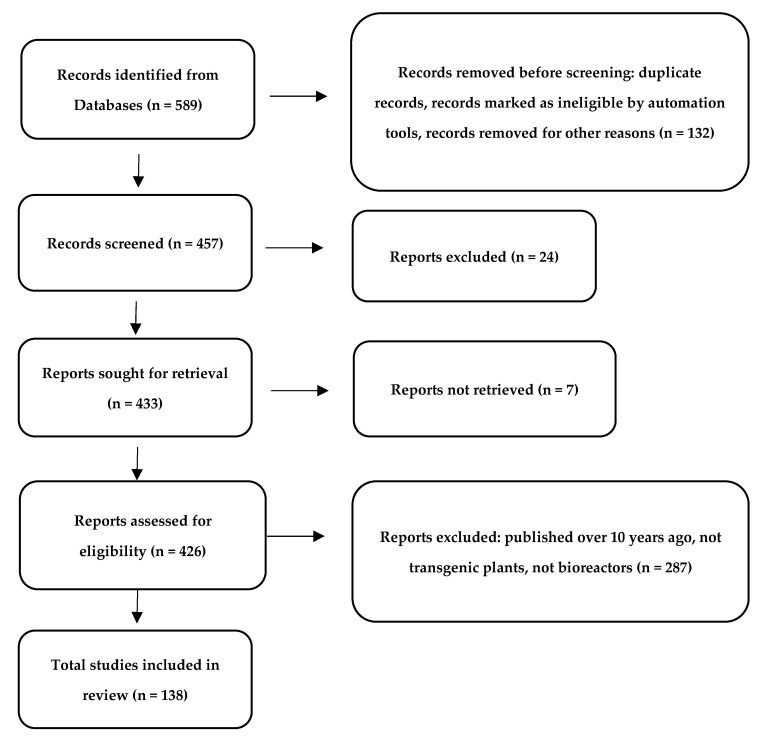
PRISMA flow diagram demonstrating the screening method for the article [32].

The present literature review was carried out using the following electronic databases: MedLars Online International Literature, PubMed, SCOPUS, and Web of Science. First, the medical subject headings (MeSH) were defined using “Solanaceae”, “plant extract”, “plant-derived compounds”, “cancer cell lines”, “anticancer effect”, ‘’chemotherapeutic with combination of plant extracts’’, and ‘’nanoparticles with plant extracts’’ as descriptive terms. The same strategy was used for all of the databases, with adaptations, as appropriate. The data presented in the tables were published in the period 2015–2021 (Figure 1).

## 3. Medicinal Plants in Cancer Treatment

Phytotherapy is believed to have originated with the Sumerian and Chinese civilizations over four thousand years ago, and became particularly prominent in ancient Egypt. The first plant to be used against cancer may have been *Trifolium pratense*: Dioscurides reported its use in the treatment of neoplasms in *De Materia Medica*, together with an extract of *Colchicum autumnale* known to have anticancer properties, which were attributed to its colchicine content. Elsewhere, Avicenna also mentions the use of *Ricinus communis*, *Atropa belladonna*, *Urtica dioica*, *Narcissus poeticus*, *Scrophularia nodosa* and *Ecbalium elaterium*. Currently, the rapid development of phytochemistry has allowed the discovery of new compounds with potential medical properties [6,33,34,35,36,37,38]. Other families of great medical importance include the Poaceae, Fabaceae, Apiaceae, Lamiaceae, Brassicaceae, Papaveraceae, Plantaginaceae, and Solananceae, etc. [39,40]. For example, in the Poaceae, *Oryza sativa* exerts anticancer effects due the presence of anthocyanins and some phenols, e.g., tricin, which can be used to treat breast cancer [41]. In turn, *Arachis hypogaea*, of the Fabaceae family, has demonstrated efficacy in the treatment of colon, prostate and breast cancer, which was associated with its β-sitosterol and sterol content [42]. In addition, *Senna obtusifolia* extract, which is rich in betulinic acid, showed cytotoxic effects in human glioblastoma U87MG and leukemic NALM6 cells [19,43]. *Daucus carota*, a member of the Apiaceae family, contains bioactive carotenoids (beta-carotene and lutein), polyacetylenes, falcarindiol and falcarindiol-3-acetate, which may be effective in the treatment of leukemia [44]. In addition, *Leonurus sibiricus* or *Leonotis nepetifolia*, of the Lamiaceae, containing phenolic acids and flavonoids, were found to demonstrate cytotoxic effects against breast, leukemia, and human glioblastoma cancer cell lines [21,24,45]. In the Brassicaceae, *Brassica oleracea* exerts anticancer effects which have been attributed to the presence of sulforaphane, which can be used to decrease prostate specific antigen (PSA) levels [46]. In addition, alkaloid-rich *Papaver somniferum* extract was proved to have a cytotoxic effect against various cancer cell lines [47]. In turn, *Plantago lanceolata* extract, of the Plantaginaceae, has demonstrated a cytotoxic effect against several breast cancer cell lines, which has been associated with its flavonoid glycosides content [48]. Patients with benign prostatic hyperplasia treated with *Solanum lycopersicum* (of the Solanaceae family) with *Serenoa repens*, lycopene and bromelain exhibited an improvement in their lower urinary tract symptom-related quality of life [49]. 

The drugs used in anticancer therapy are mainly based on limiting the division of pathological cells and inducing apoptosis. The currently used anticancer cytostatic agents most often induce programmed cell death, damaging DNA. The use of natural compounds with potential anticancer activity seems interesting due to the fact that it may open up new possibilities for their use in the development of more effective methods of cancer therapy. Their antitumour activity is often based on a complex mechanism including antioxidant activity, carcinogen inactivation, antiproliferation, cell cycle arrest, the induction of apoptosis and differentiation, the inhibition of angiogenesis, and the abolition of multidrug resistance [50,51,52,53].

Cancer treatment modalities are generally based on combinations of chemotherapy, radiotherapy, surgery, hormone therapy, immunotherapy and targeted drug therapy. Chemotherapy is an important option in the treatment of cancer, and plant-derived chemotherapeutic agents have contributed significantly to advances in its development [54,55,56,57,58,59,60,61,62,63]. A number of clinically-applied agents from plant sources were standard ingredients in many anticancer therapies, including vinblastine, vincristine (*Catharanthus* sp.), paclitaxel (*Taxus* sp.), camptothecin derivatives (*Camptotheca* sp.) and podophyllotoxins (*Podophyllum* sp.) [9,63,64,65,66,67]. Currently, vincristine encapsulated in liposomes is approved for the treatment of acute lymphoblastic leukemia, while protein-bound paclitaxel particles are approved for the treatment of non-small cell lung cancer, according to a Food and Drug Administration (FDA) report [68]. It is noteworthy that, in some cases, their use is associated with various side effects that might limit their broad clinical use [69]. 

Plant compounds generally suffer from low bioavailability and hydrophobicity issues, which have been addressed with the use of recently discovered nanomaterials, many of which may also be of natural origin. Nanoparticle application has led to the increase of the concentration of the drug in neoplastic cells with particular receptors on their surface [70,71,72,73,74]. A nanoparticle consists of a drug on the inside, and a so-called “ligand”—i.e., a molecule designed to bind to a tumour cell receptor—on the outside. After binding to the receptor, the nanoparticles are absorbed into the cell and the drug is released. Compounds of plant origin (pure compounds or extracts) have been used in combination with nanoparticles and with chemotherapeutic agents in adjunctive therapies [75,76]. 

However, it should be noted that plant preparations have more complicated and unpredictable interactions with drugs than would be expected between two conventional drugs due to the numerous active compounds found in the plant raw materials. This may be due to the fact that compounds of plant origin may influence the pharmacokinetics and pharmacodynamics of the anticancer drugs used. As a consequence, toxic drug effects may be observed or treatment efficacy may be reduced [77,78,79,80]. So far, numerous studies have highlighted the positive effects deriving from the co-administration of drugs with plant extracts. For example, Hussain et al. reported a synergistic effect between cisplatin and *Aloe vera* extract on MCF-7 and HeLa cancer cell lines, suggesting that the plant extract may increase the therapeutic efficacy of conventional anticancer drugs [81]. In addition, *Senna obtusifolia* extract was found to have a synergistic effect in combination with doxorubicin [43].

## 4. Nanotechnology and Plant Compounds in the Fight against Cancer

The advance of modern technology has brought new products and research techniques which have driven significant scientific progress. Nanotechnologies have successfully entered everyday life, and are increasingly used in medical sciences. Many common devices are based on the achievements of modern nanotechnology, such as energy-efficient and powerful electronic devices, versatile nanocoatings, and new-generation cosmetics [82,83,84]. In its simplest definition, nanotechnology is a “nano-scale technology”, i.e., a technology in the size range 1–100 nm. For a better understanding of these sizes, the nanometer scale (nm) is one billionth of a meter, or three to five atoms wide: less than one tenth of a micrometer in at least one dimension. 

The latest scientific achievements may turn out to be effective treatments for diseases that have been troubling people for centuries, among which cancer is still a challenging and often unresolved issue. In the past, the detection of neoplastic diseases was possible only after the appearance of specific symptoms, generally in an advanced stage of the disease, and even in the presence of distant metastases. A more complete view of pathologically-changed tissues can be obtained by biopsy; however, this method has many limitations, and carries the risk of complications [85,86]. In response, highly-sensitive non-invasive methods of detecting neoplastic diseases have been developed, most of which rely heavily on nanotechnology [87]. 

In fact, nanomaterials are widely used in the diagnosis and treatment of different types of cancer due to the possibility of precisely controlling their shapes, sizes and specific physical properties. Nanoparticles can also serve as carriers of anticancer drugs to specific cells [88], and nanomaterial-based devices used to detect the proteins or nucleic acids of cancer cells can provide an early indication of disease or monitor the effectiveness of therapy. Such biomarkers can be detected in body fluids such as blood, saliva and urine. One such group of tumour biomarkers are proteins. A number of biomarkers are routinely tested in clinical practice—PSA (prostate cancer), CEA (colorectal cancer), CA-125 (ovarian cancer), ER (breast cancer), AFP (liver cancer) and CA 19-9 (pancreatic cancer)—and nanosensors can be successfully used to detect them [89,90,91,92,93,94]. The most frequently used nanoparticles in the diagnosis of cancer diseases are gold nanoparticles, nanoshells and quantum dots [95,96]. 

Nanoparticles such as micelles, dendrimers, quantum dots, liposomes and carbon nanotubes can also be used in the treatment of neoplastic diseases. Traditional chemotherapeutics include alkylating agents and antibiotics that induce damage to the DNA of cancer cells. Topoisomerase or mitosis inhibitors are also used [97]. Many of these therapeutics are highly effective, but they often demonstrate a lack of specificity, resulting in severe side effects [98]. There is a clear need for new methods allowing for the effective and specific targeting of neoplastic cells. One potential strategy that has received much attention over the past few years involves the use of nanoparticles [99,100,101], as well as those based on a combination of modern nanotechnology with a rich arsenal of compounds of natural origin with anticancer properties [102,103,104,105,106]. 

The nanoparticles themselves are typically obtained by electrospraying, evaporation–condensation, laser ablation or pyrolysis, or high-energy ball milling. They can also be obtained chemically by chemical vapor synthesis, the sol-gel method, hydrothermal synthesis, microemulsion technique, or polyol synthesis [107,108]. It is worth emphasizing that physical methods sometimes have an advantage over chemical methods due to the lack of danger of solvent contamination in the prepared thin films, and due to the uniformity of the synthesized nanoparticle distribution [109]. However, these synthesis methods are often complicated and require strictly controlled temperature, pH and pressure conditions, as well as specialized equipment, and often environmentally-hazardous reagents containing heavy metals [110]. Hence, many research teams are interested in the biological synthesis of nanoparticles, which should offer weak contamination with toxic agents, the customization of desired properties, repeatability and easy scalability [111,112]. 

Among the various biological systems used for this purpose, plants deserve special attention because plant cells may contain a wide range of bioactive compounds with potential anticancer properties. One study examined the antitumour potential of *Nepeta deflersiana* extract in silver nanoparticles (ND-AgNPs) against human cervical cancer (HeLa) cells, as well as the influence of cytotoxic concentrations of ND-AgNP on markers of oxidative stress, reactive oxygen species (ROS) production, mitochondrial membrane potential, cell cycle arrest, and apoptosis/necrosis. It was found that the cytotoxicity of the tested particles was concentration dependent, and that the treatment was associated with a significant increase in ROS and lipid peroxidation, and a decrease in matrix metalloproteinases (MMPs) and glutathione levels. The cell cycle analysis and apoptosis/necrosis assay data showed that ND-AgNP induced SubG1 arrest and apoptotic/necrotic cell death [113]. Gomathi et al. examined the potential for silver nanoparticles to be biosynthesized in the fruit shell of *Tamarindus indica*. Here, too, the plant extract acts as a reducing and stabilizing agent for silver nanoparticles. These nanoparticles proved to be cytotoxic to MCF-7 cell lines; hence, they could be considered as potential therapeutic agents in the treatment of human breast cancer [114]. 

**Figure 2:**
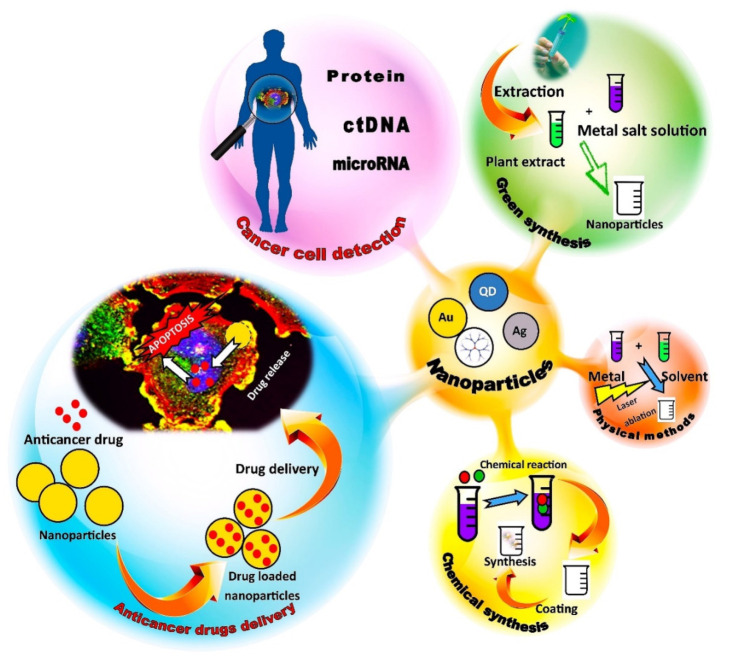
General scheme of the synthesis and application of nanoparticles in cancer diagnosis and treatment.

Because plants from the Solanaceae family produce a number of compounds with proven or potential anticancer activity, they may well be used on a large scale as the basis for new systems for the biosynthesis of nanoparticles exhibiting anticancer activity. Combined with the extremely rapid technological progress, this may be the starting point for the development and implementation of completely new and more effective methods of fighting cancer. The general scheme of the synthesis and application of nanoparticles in cancer diagnosis and treatment is presented in Figure 2.

## 5. Synergy between Chemotherapeutic Agents and Plant Compounds in Cancer Therapy

Despite its many side effects, chemotherapy remains the most popular treatment for cancer. Although many chemotherapeutic compounds are of plant origin (e.g., paclitaxel, camptothecin, colchicine, vincristine, and podophyllotoxin, etc.), being either synthetic or isolated directly from plants, they have considerable side effects. Moreover, their low water solubility, poor penetration into target cells, limited therapeutic potential and toxic side effects may limit the suitability of these natural agents for the treatment of cancer [9,11,63,115,116,117]. Therefore, new phytochemical anticancer agents require substantial evidence of efficacy from appropriate preclinical trials before their approval for use in patients [79,118,119,120,121]. It is also possible to chemically modify the molecule and improve its properties [122].

One new therapeutic strategy which is based on the synergistic action between chemotherapeutic agents and plant compounds intends to overcome these shortcomings. Synergy comes from the Greek word “*synergos*”, which means “working together”, and is broadly defined as the interaction of two or more compounds or other factors to produce a combined effect greater than the sum of their separate parts [123,124]. Synergistic effects are believed to arise from synergistic multi-target effects, the modulation of pharmacokinetic or physicochemical effects, interference with resistance mechanisms, or elimination and neutralization potentials [125,126,127,128]. Studies show that a secondary compound or plant extract—such as essential oil derivatives, polyphenol derivatives or terpenoid derivatives—may be capable of removing or neutralizing the toxic effects or side effects of a drug [123,129,130,131].

## 6. Solanaceae Family—General Characteristics and Application

**Figure 3:**
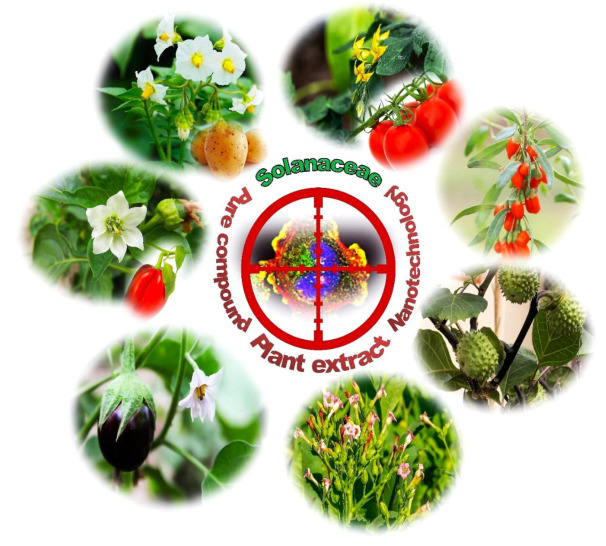
Selected examples of species from the Solanaceae family exhibiting anticancer activity, such as *Solanum tuberosum*, *Capsicum annuum*, *Solanum melongena*, *Lycopersicon esulentum*, *Nicotiana tabacum*, *Datura stramonium* and *Lycium barbarum*.

The Solanaceae (nightshades) are considered to be the third most economically-important family in the plant kingdom after the Poaceae and the Fabaceae. They are also one of the most significant families of trees, shrubs and herbs, with great floristic, phytochemical and ethnobotanical importance, with over 90 genera comprising 3000–4000 species spread all over the world. Almost half of these belong to the large and varied genus *Solanum*. It is distributed in all continents except Antarctica, with the greatest diversity being observed in Central and South America [132,133,134,135,136]. In addition to *Solanum*, the leading genera of the Solanaceae family include *Atropa*, *Datura*, *Capsicum*, *Nicotiana*, *Lycium*, *Hyoscyamus*, *Lycopersicon*, *Withania* and *Petunia*. This single-genus hyper-diversity is remarkable in angiosperms, making *Solanum* interesting from an evolutionary point of view, as well as for its usefulness to humans (Figure 3). 

The representatives of the Solanaceae vary extremely in regards to their habit, distribution and morphology, with an astonishing variety of flowers and fruits. The flowers are usually radially symmetrical, with five united sepals, five united petals, five stamens inserted on the tube. The ovary is positioned superior. It consists of two united carpels with the partition walls often present, but more obvious in wild species than domestics. The leaves are alternate, or rarely opposite, and are usually simple. The fruit is a two-chambered capsule called a berry [28,29,137,138,139,140,141,142,143]. In addition to a wide range of other uses (e.g., traditional medicine, traditional culture, pharmacology, and ornamental horticulture), the species of the Solanaceae are of great importance as food crops around the world. In 2020, the global areas cultivated with four basic species— potatoes, tomatoes, aubergines and capsicums (chilies and green peppers)—were 16.5, 5.1, 1.9 and 2.1 million hectares, with productions of 359.1, 186.8, 56.6 and 36.1 million tons, respectively [144]. Moreover, from a biotechnological point of view, species from the seven genera of the Solanaceae have become the subject of genetic research as model plants and/or because of their importance as crops. Model plants include cultivated tomatoes and their wild relatives (genus *Solanum*, former genus *Lycopersicon*), tobacco (genus *Nicotiana*), and species of petunias (genus *Petunia*) [29,142,143]

The Solanaceae are also known to possess a diverse range of biologically-active compounds that can be used to benefit human health, such as phenolics, alkaloids, saponins, terpenes and lipids. However, toxic alkaloids such as tropane alkaloids or glycoalkaloids are of particular interest because of their reported antimicrobial, anti-rheumatic and antioxidant activities [145]. They have also demonstrated antitumour activity against several types of cancer, including prostate, breast and colon cancer [146,147]. Tropane alkaloids such as atropine, hyoscyamine and scopolamine have a characteristic bicyclic structure, and particularly high concentrations have been found in *Datura stramonium*, *Datura ferox* and *Datura innoxia.* Atropine is a racemic mixture of two enantiomeric forms of hyoscyamine, with the L-enantiomeric form being the active one. Scopolamine, which acts as an antagonist at both the peripheral and central muscarinic receptors, is the most valuable member of a group known as the tropane alkaloids [143,147,148]. This group is highly diverse, being formed from a tropane skeleton, which is highly prone to modification. Tropane alkaloids are found in all plant parts, with the highest concentrations in roots and seeds. Their levels vary according to their species, season, location, and plant organ. From a pharmacological standpoint, they are well known as potent anticholinergic agents, meaning that they inhibit neurological signals from the endogenous neurotransmitter acetylcholine. The symptoms of an overdose may include a dry mouth, ataxia, dilated pupils, convulsions, urinary retention, hallucinations, coma, and death [140,147,149].

Glycoalkaloids are produced in more than 350 plant species, particularly those of the Solanaceae and Liliaceae families. They are a group of glycosidic derivatives of nitrogen-containing steroids consisting of a cholestane skeleton with a carbohydrate moiety of one to five monosaccharides attached at the 3-OH position [150]. Arguably, the most significant glycoalkaloids are α-solanine and α-chaconin, which are contained in potatoes (*Solanum tuberosum*); solasonin and solamargine, in eggplants (*Solanum melongena*); and α-tomatin and dehydrotomatin, which are spirosolan-type glycoalkaloids found in tomato plants (*Lycopersicon esculentum*) [151].

## 7. Anticancer Effect and Potential Mechanisms of Action of Plant Extracts from the Solanaceae Family

Thanks to their wide range of active substances, plant extracts exert a variety of effects on cancer cells, with some of them having been reported to have inhibitory effects on cell proliferation [152]. Indeed, a number of in vitro and in vivo studies have found extracts from Solanaceae family members to also have strong anticancer properties. 

In the *Solanum* genus, a *Solanum lycopersicum* leaf extract was shown to exhibit potential antitumour properties against breast cancer cells by modulating the expression of genes associated with cancer growth and progression [153]. In addition, a *Solanum lyratum* extract taken from the whole plant exhibited a proapoptotic effect against human osteosarcoma epithelial cells. The apoptosis induction took place through a number of routes: the increase of reactive oxygen and nitrogen species production; the decrease of mitochondrial membrane potential; the release of cytochrome c; the activation of caspase 3, 8 and 9; the increase of the level of proapoptotic proteins, including Bax; and the decrease of the level of anti-apoptotic proteins, including Bcl-2 [154]. *Solanum nigrum* fruit extract has also been found to decrease viability by the induction of apoptosis and cell cycle arrest at the G2/M phase in prostate cancer cells [155], and to inhibit the proliferation, migration and invasion of glioma cells by the induction of their apoptosis [156].

Extracts of *Withania* species are also indicated to have specific cytotoxic properties against cancer cells. *Withania somnifera* leaf extract was found to have cytotoxic effects against human osteosarcoma, fibrosarcoma and lung cancer epithelial cells, and to activate tumour suppressor proteins including p53 [157]. Similarly, extracts from different parts of *Withania coagulans* were found to bestow antiproliferative properties and NFκB pathway induction [158]. 

In addition, *Capsicum annuum* seed extract has been shown to inhibit the migration of lung cancer and breast cancer cells by downregulating metalloproteinases MMP-2 and MMP-9, and increasing E-cadherin expression [159]. Furthermore, leaf and shoot extracts of *Nicotiana glauca* exhibit cytotoxic properties against lung cancer and prostate cancer cells, and demonstrate anti-angiogenic properties in vivo by inhibiting microvessel formation [160].

Recent reports on the anticancer properties of plant extracts in the Solanaceae family are listed in Table 1.

## 8. Anticancer Effect and Potential Mechanisms of Action of Pure Compounds Isolated from the Solanaceae Family

Although plants and natural extracts are very important sources of biologically-active compounds, the study of their isolated products can provide a starting point for the development of new drug candidates with unique structures and mechanisms of action. Indeed, many of the secondary metabolites produced by the Solanaceae have been found to be of medical importance, with effects on cancer cells [13].

For example, solajiangxins H, solajiangxins I and 7-hydroxylsolajiangxin I isolated from whole plant extracts of *Solanum lyratum* show cytotoxic effect against intestinal cancer cells [213]. *Solanum aculeastrum*, containing steroidal glycosides, showed antitumour activity against various cancer cell lines, including lung, colon and cervical cancer cells [214]. In turn, Shieh et al. demonstrated the time- and dose-dependent inhibition of cell viability in α-tomatine-treated non-lung cancer cells [215]. The steroidal alkaloid soladulcidine, isolated from *Solanum dulcamara*, and ten of its derivatives were shown to have significant antiproliferative effects against prostate cancer cells [216]. In addition, 35 withanolides and withaferin A from the roots and leaves of *Withania somnifera* have demonstrated efficacy against a wide range of cell lines [217,218]. Withawrightolide and four other withanolides derived from the aerial parts of *Datura wrightii* were similarly found to exhibit cytotoxic properties against glioma cells [219]. In addition, Physalis peruviana seed extract induced apoptosis in HeLa cells [220].

In particular, secondary metabolites of the Solanaceae family are known to induce apoptosis in various types of cancer cells by activating different signalling pathways. These differences may result from both chemical structure of the compounds and specific sensitivity of cancer cells. Such compounds with antiproliferative properties commonly affect processes associated with the cell cycle, gene expression, signal transduction pathways, changes in the mitochondrial membrane, metabolic pathways, and autophagy [31].

The cell cycle is an important mechanism that determines cell proliferation. Alkaloids such as baimantuoluoamide A and baimantuoluoamide B inhibit cyclin-dependent kinase 4 (CDK4) activity, and glycoalkaloids such as solasonine, solanidine, and solamargine induce cycle arrest in the S phase. Arrest in the G2/M phase is induced by solamargine and withaferin A [24]. Withaphysalin F—isolated from the leaves of *Acnistus arborescens*—also has anti-proliferative properties and the ability to arrest the cell cycle in the G2/M phase, which has been attributed to the inhibition of tubulin polymerization and the induction of DNA fragmentation [221].

The compounds also influence gene expression. For example, withaferin A is known to inhibit transcription factors such as MYB and C/EBPβ [31]. In addition, solasonine, β1-solasonine, solamargine and solanigroside P isolated from the aerial part of *Solanum nigrum* show antiproliferative properties against gastric cancer cells, and can induce apoptosis by altering gene expression, such as by increasing Bax expression, decreasing Bcl-2 expression, and activating caspase-3 [222]. Lycopene, a carotenoid found commonly in *Solanum lycopersicum,* was found to regulate the expression of various apoptosis-related proteins and genes—such as caspase-3, caspase-8, Bax, Bax:Bcl-2 and Bcl-xL—among breast cancer cells [223]. Furthermore, physalin F derived from the whole plant *Physalis minima* has cytotoxic effects and induces the apoptosis of breast cancer cells through caspase-3 activation and DNA fragmentation [224]. In turn, solamargine increased the expression of p53, Bax and Bcl-2 in U2OS and K562/A02 cells on the mRNA and protein levels, and the mRNA expression and promoter activity of EP4, as well as the protein expression of SP1 and NF-κB subunit p65 in lung cancer cell lines [225].

In addition, they can also inhibit various signalling pathways that may be responsible for cell growth and proliferation. For example, withanolide S5 inhibits receptor tyrosine kinases, withametelin and coagulansin A downregulate the Mitogen-Activated Protein Kinase (MAPK) pathway and the phosphatidylinositol-3-kinase (PI3K) pathway, and 4β-hydroxywithanolide E targets the Wnt/β-catenin pathway. Solamargine suppresses the phosphorylation of Akt [225]. In addition, withaferin A inhibits colon cancer by inhibiting Notch-1 signalling, as indicated by the downregulation of Notch-1 targets including Hes-1 and Hey-1; it also inhibits its cross-talk with the Akt/mTOR pathway, thus suggesting the Notch-Akt-mTOR axis as a therapeutic target in colon cancer [226]. Furthermore, α-chaconine and α-solanine reduce the expression and activity of the Akt and ERα signalling pathways in human endometrial carcinoma cells [227]. In addition, arabinogalactan upregulates two of the three MAPK cascades, including c-jun N-terminal kinase (JNK) and p38 kinases, and downregulates the third based on extracellular signal-regulated kinases (ERK), and scopoletin demonstrates a strong binding affinity with vascular endothelial growth factor (VEGFA), which is involved in signalling [31].

Solanaceae-isolated compounds may also induce apoptosis by influencing the mitochondrial membrane. Defensin (NoD173), for example, permeates the mitochondrial outer membrane, resulting in the potential collapse of the membrane, followed by the release of cytochrome c and the activation of caspases. In turn, α-solanine was found to induce mitochondrial mediated apoptosis by opening pores and inducing the release of cytochrome c and Smac from mitochondria into the cytosol, further activating caspase-9 and decreasing the mitochondrial membrane potential [228].

They may also alter metabolic pathways: physapubescin I blocks kidney-type glutaminase, an enzyme involved in ATP production. Its downregulation may inhibit the growth and proliferation of cancer cells. Finally, physapubescin B is known to activate autophagy via mTORC1 inhibition, while physapubenolide downregulates key proteins involved in the process [31].

Recent reports on the anticancer properties of pure compounds isolated from plants from the Solanaceae family are listed in Table 2.

**Figure 4:**
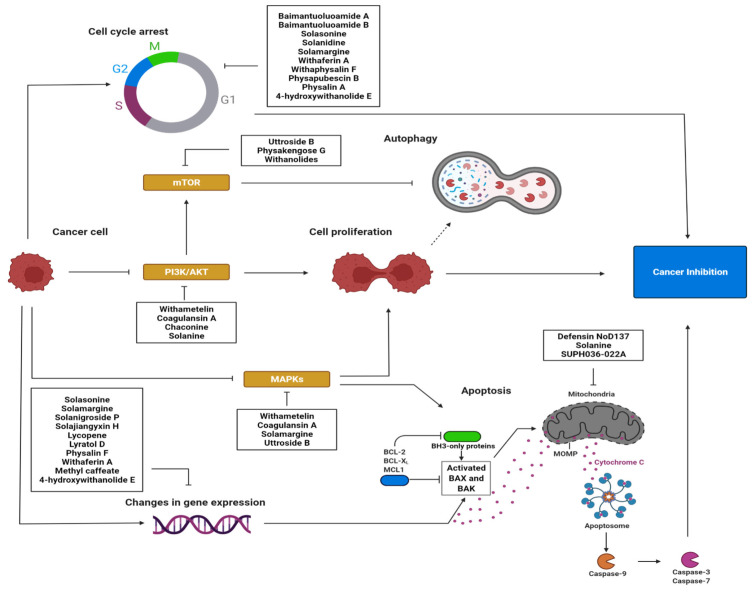
Schematic diagram presenting the potential anticancer effect of compounds from the Solanaceae family through the induction of apoptosis and the activation of signalling pathways in cancer cells (created using BioRender).

The mechanisms of action described in the text and included in Table 2 are presented in Figure 4.

## 9. Anticancer Effect and Potential Mechanisms of Action of Nanoparticles in Combination with Plant Extracts from the Solanaceae Family

Plant-based nanomaterial synthesis has been growing in popularity. The approach is more environmentally friendly than chemical or physical methods, and many studies have reported that it yields nanoparticles with improved pharmacological properties [277]. Regarding extracts from the Solanaceae, the available data indicate that silver nanoparticles generated by *Datura inoxia* exert significant antiproliferative effect against lung cancer cells. They also induce apoptosis cell cycle arrest and inhibit DNA synthesis [278]. Recent reports on the anticancer properties of nanoparticles using extracts from the Solanaceae family are listed in Table 3.

## 10. Synergistic Effect of Chemotherapeutic Drugs and Plant Extracts from the Solanaceae Family

The occurrence of drug resistance indicates the need to search for new chemotherapeutic agents and improved combinations of them. Combined anticancer therapy uses drugs that target different pathways, as this can result in improved cytotoxicity for the cancer cell, with both additive and synergistic effects [293]. Synergy can also be observed between conventional drugs and chemical compounds and extracts, and some plant-derived compounds have been found to improve the effeciency of anticancer therapy [124]. For example, *Solanum nigrum* leaf extract has been found to enhance the effect of cisplatin, doxorubicin, docetaxel and 5-fluorouracil, resulting in the induction of intestinal cancer cell autophagy through the accumulation of microtubule-associated proteins [294]. Moreover, whole-plant *Solanum nigrum* extract has intensified the effect of doxorubicin in the suppression of the growth of HeLa [295] and breast cancer cells [296]. Recent reports on the synergistic properties of extracts from the Solanaceae family and anticancer drugs are listed in Table 4.

## 11. Anticancer Effect in In Vivo Studies of Compounds of the Solanaceae Family

Due to due to their high content of bioactive compounds, Solanaceae family members have also been used in a number of in vivo studies. This review categorizes in vivo studies as extracts, pure compounds, nanoparticle extracts and chemotherapeutic extracts. For example, Wu et al. showed that SR-T100—extracted from *Solanum incanum* (solamargine alkaloid)—caused all papillomas (35/35) and 27 of the 30 UVB-induced microinvasive squamous cell carcinoma in hairless mice to disappear within 10 weeks of the daily use of topical SR-T100 [304]. In other studies, Wu et al. found that *Solanum incanum* extract (SR-T100) paclitaxel and cisplatin inhibited the growth of A2780CP70 cells in mouse xenografts, compared to the vehicle control, and that the combination of cisplatin and SR-T100 was more effective than either treatment alone. The authors suspect that SR-T100 may represent a potential therapeutic adjunct to chemotherapy for ovarian cancer treatment [300]. Furthermore, *Solanum lyratum* aerial part extract was found to significantly inhibit the growth of S180 sarcoma in mice in vivo, and to increase the proliferation of splenocytes, natural killer cells and cytotoxic T cells, as well as interleukin 2 and interferon-γ, by splenocytes. The authors propose that the extract exhibits its anti-tumour effects through its immunomodulatory properties [305]. Solasodine and rhamnosyl glycosides isolated from *Solanum sodomaeum* were found to demonstrate antitumour properties in a mouse model [306].

In turn, Deng et al. revealed that the fraction from *Lycium barbarum* polysaccharide could reduce immunotoxicity and enhance the antitumour activity of doxorubicin in mice. The results showed that *Lycium barbarum* polysaccharide did not protect against the bodyweight loss caused by doxorubicin, but it promoted the recovery of bodyweight starting at day 5 after doxorubicin treatment in tumour-free mice. *Lycium barbarum* polysaccharide also improved peripheral blood lymphocyte counts, promoted cell cycle recovery in bone marrow cells, and restored the cytotoxicity of natural killer cells. Furthermore, in H22 tumour-bearing mice, *Lycium barbarum* polysaccharide enhanced the antitumour activity of doxorubicin, and improved the peripheral blood lymphocyte counts and the cytotoxicity of splenocytes [307]. Diwanay et al. noted that the alkaloid-free polar fraction of *Withania somnifera* resulted in protection towards cyclophosphamide-induced myelo- and immunoprotection, as was evident from the significant increase in white cell counts and hemagglutinating and hemolytic antibody titers. Treatment with these candidate drugs may be important in the development of adjunctive therapy with anticancer chemotherapy [308].

The anticancer and radio-sensitizing efficacy of a *Withania somnifera* extract/Gadolinium III oxide nanocomposite (WSGNC) was also investigated in mice. WSGNC treatment combined with γ-radiation led to a significant decrease in the solid Ehrlich carcinoma size and weight in mice; this was associated with a significant decrease in mitochondrial enzyme activities, glutathione content and superoxide dismutase (SOD) activity, as well as a significant increase in caspase-3 activity, malondialdehyde concentration and DNA fragmentation in cancer tissues. The authors indicate that WSGNC can be considered as a radio-sensitizer and an anticancer modulator, suggesting a possible role in the reduction of the radiation exposure dose during radiotherapy [309]. Further studies are presented in the Table 5.

## 12. Conclusions and Future Perspectives

Cancer is a devastating disease, and the currently available treatments for patients are generally associated with undesirable adverse effects. The use of medicinal plants to manage or arrest the carcinogenic process provides an additional strategy that can be used alongside treatments with canonical drugs. Many plant-derived bioactive compounds have achieved favorable results in clinical studies, and their tumouricidal properties against various cancers are under investigation.

This literature review evaluated the anticancer properties of natural products from the Solanaceae family. They were grouped in terms of extracts, pure compounds, nanoparticles with extracts, and chemotherapeutic agents with extracts, and their potential mechanisms of action were given. Although all of the studies found the extracts to demonstrate strong in vitro and in vivo anticancer activity in cancer cell lines and animal models, more research is needed in order to elucidate their specific mechanisms of action, and to determine their potential for cancer prevention and treatment.

Plants of the Solanaceae family are widely discussed due to their multi-directional activity. Multiple in vitro studies have been reported with promising results. On the other hand, the anti-tumour potential of the secondary metabolites from Solanaceae is also quite clear. In addition, nanotechnology techniques can enhance their action and eliminate negative effects on normal cells. Thus, plants of the Solanaceae family should be tested further in order to better elucidate their therapeutic potential not only in in vitro and in vivo studies but also in clinical applications. However, the study of these plants should not limit the study of the plethora of anticancer plants, some of which are still unexplored. Research is needed in order to elucidate the antitumour mechanism of action of many already studied and unexplored plants.

## Figures and Tables

**Table 1:** In vitro anticancer effect of plant extracts from the Solanaceae family and their potential mechanisms of action.

Name of the Species	Part of the Plant	Type of Solvent	Class of Compounds/Compounds Identified in Extract/Fraction	Cancer Cell Lines	Activity/Mechanism/Effect	Ref.
*Athenaea velutina* Sendtn.	leaves	dichloromethane: methanol (1:1)	phenolic compounds and flavonoids	MCF-7, HepG2, B16-F10	Cytotoxic (IC_50_ values in the range of 1.56–200 μg/mL) (MTT test); inhibition of migration, adhesion, invasion and cell colony formation.	[161]
*Capsicum annuum* L.	red pericarp	water/methanol	capsianoside derivatives	HCT116, PC-3	Cytotoxic (IC_50_ = 51 μg/mL and 60 μg/mL) (MTT test)	[162]
*Capsicum annuum* L.	fruits	ethanol	carotenoids, chlorophyll, polyphenols, tannins, and flavonoids	Calu6	Cytotoxic	[163]
*Solanum betaceum* Cav.	fruits	ethanol	phenolics	HepG2, MDA-MB-231	Cytotoxic (IC_50_ values in the range of 30–80 μg/mL) (MTT test)	[164]
*Datura innoxia* Mill.	leaves	water	phenolic and flavonoid contents	K562	Cytotoxic (IC_50_ = 0.6 mg/mL) (MTT test); antiproliferative activity by interaction with DNA and histones	[165]
*Datura stramonium* L., *Datura inoxia* Mill.	leaves	ethyl acetate	rutin, gallic acid, catechin, apigenin and caffeic acid	PC-3, MDA-MB 231, MCF-7	Cytotoxic (IC_50_ < 3 μg/mL) (MTT assay); anti-tumour activity (evaluation of haematological, biochemical and histological)	[166]
*Hyoscyamus reticulatus* L., *Hyoscyamus tenuicaulis* Schönb.-Tem. *Lycium shawii* Roem. & Schult. and *Solanum luteum* L.	shoots, leaves, stems	dichloromethane	-	MOLT-4	Cytotoxic (IC_50_ values in the range of 35.5–>50 μg/mL) (MTT test)	[167]
*Ipomoea batatas* (L.) Lam.	root tubers and leaves	methanol/trifluoroacetic acid (TFA), ethanol/TFA, methanol/TFA/water, and ethanol/TFA/water	anthocyanins	MCF-7, HCT-116, and HeLa	Antiproliferative properties	[168]
*Lycium barbarum* L.	fruits (Goji berries)	ethanol	-	T47D	Cytotoxic (IC_50_ = 0.75 mg/mL) (MTT test); induction of apoptosis by changes of the apoptotic protein expression (increase in pro-apoptotic proteins and a decrease in anti-apoptotic proteins)	[169]
*Lycium barbarum* L.	fruits	-	phenolics	HepG2	Cytotoxic (18%, at 1600 μg/mL) (MTT test)	[170]
*Lycium barbarum* L.	fruits	methanol/ethyl acetate/petroleum ether	zeaxanthin-rich extract	BJ HEP, A375	Cytotoxic (IC_50_ = 75.15 and 85.06 μM for BJ HEP, 62.36 and 92.59 μM for A375)	[171]
*Lycium barbarum* L.		water	pectin-free, polysaccharides fraction	MCF-10A, MCF-7, HER2, MDA-MB-231	Cytotoxic 1000 μg/mL (MTT test)	[172]
*Lycium barbarum* L.	fruits	-	carotenoids	Caco-2 cells	Effect (range from 6.5 to 92.8%) (MTT test)	[173]
*Lycium barbarum* L., *Lycium ruthenicum* Murr	fruits	ethyl acetate	phenolics flavonoids, carotenoids	MDA	Cytotoxic (EC_50_ = 4.08 mg/mL); apoptosis via modulating cell cycle arrest, cell apoptosis, and the p53 signalling pathway	[174]
*Lycium chinense* Mill.	fruit (Goji berries)	ethanol	-	LS180	Cytotoxic (MTT test)	[175]
*Lycium europaeum* L.	fruit	methanol	phenolic, flavonoids, anthocyanins, carotenoids, lycopens, and condensed tannins content	A549, PC12	Cytotoxic (MTT assay), morphological changes and induction of apoptosis by caspase 3/7 activation	[176]
*Lycopersicon esculentum* Mill.	leaves	hydromethanol, acetone and alkaloid extracts	phenolic compounds, pigments, and alkaloids	AGS	Cytotoxic (IC_50_ values in the range of 9–171 μg/ mL)	[177]
*Nicotiana glauca* Graham	leaves	ethanol	palmitic acid and scopoletin	CCL-136	Anti-proliferative effect and induction of apoptosis by changes in mitochondrial and nuclear morphology	[178]
*Nicotiana glauca* Graham	stem	n-hexane	beta-sitosterol, stigmasterol, campesterol, D-alpha-tocopherol, scopoletin, 3,7,11,15-tetramethyl-2-hexadecen-1-ol, Bicyclo[3.1.1]heptanes	MCF-7	Cytotoxic (IC_50_ = 17.98 μg/mL) (MTT test); induction of apoptosis by changes in mitochondrial membrane potential, chromatin condensation and cytoplasmic shrinkage	[179]
*Physalisalkekengi* L.	fruit	chloroform	physalin D	HeLa MCF-7, A431	Growth inhibition	[180]
*Physalis angulata* L.	leaves	ethanol	-	SKOV3, HL-60	Cytotoxic (IC_50_ in the range of 18–375 μg/mL) (MTS test)	[181]
*Physalis peruviana* L.	fruit	ethanol, isopropanol	ursolic acid, rosmarinic acid, gallic acid, quercetin, and epicatechin	HeLa	Cytotoxic (IC_50_ = 60.48 μg/mL) (Resazurin Reduction)	[182]
*Solanum aculeastrum* Dunal	whole plant	methanol	solamargine and solanine	SH-SY5Y	Cytotoxic (IC_50_ = 10.72 μg/mL) (sulforhodamine B (SRB) colorimetric assay)	[183]
*Solanum capsicoides* All.	seeds	methanol	carpesterol	K562	Cytotoxic (U251 GI50 = 24.7 μg/mL, MCF-7 GI50 = 27.1 μg/mL, 786-0 GI50 = 25.8 μg/mL, OVCAR-03 GI50 = 24.0 μg/mL, and K562 GI50 = 32.0 μg/mL) (Toxicity Estimation Software Tool (TEST) software)	[184]
*Solanum chacoense* Bitter.	leaves, tubers	methanol	phenolic acids and volatile compounds	MCF-7	Cytotoxic (IC_50_ values in the range of 132.9–390.7 μg/mL) (MTT test); induction of apoptosis by changes in expression of proliferation- and apoptosis-related genes (overexpression Bax¸ down-regulation Bcl-2)	[185]
*Solanum incanum* L.	whole plant	acid base precipitation followed by the different ratios of ethanol/H2 O extraction (according to the patents—US patent 7,078,063, EU patent 1,058,334, and Japan patent 3,940,928)	solamargine	B16	Cytotoxic (IC_50_ in the range of 2.91–6.85 μg/mL) (MTT test); induction of apoptosis by DNA damage and activation of caspase 9; G0/G1 cell cycle arrest	[186]
*Solanum incanum* L.	fruit	water	-	HCT-116	Cytotoxic (IC_50_ = 23.35 μg/mL) (Sulphorhodamine B test); ultrastructural changes (loss of the surface microvilli, mitochondrial damage, formation of autophagic vacuoles, nuclear shrinkage, chromatin condensation and nucleolar changes)	[187]
*Solanum incanum* L., *Solanum schimperianum* Hochst, *Solanum nigrum* L., *Physalis lagascae* Roem. & Schult. and *Withania somnifera* (L.) Dunal	leaves	methanol	hydroxycinnamic acid amides, steroid alkaloids, steroidal glycoalkaloid fractions	MCF-7, MDA-MB-231, HT-29, HTC-116	Cytotoxic (IC_50_ values in the range of μg/mL and 1.29–19.83 μg/mL) (MTT test)	[188]
*Solanum lycopersicum* L.	fruit	methanol	phenolic, ascorbic acids and flavonoid content	HepG2, HeLa	Cytotoxic (IC_50_ values in the range of 156–212 μg/mL) (MTT test)	[189]
*Solanum lycopersicum* L.	fruit	ethanol/water	carotenoids, phenolics, sterol content, fatty acid	HT-29	Cytotoxic (IC_50_ = 150 μg/mL) (MTT test)	[190]
*Solanum lycopersicum* L.	leaves	ethanol	-	A549, HeLa	Cytotoxic (IC_50_ < 31.25 μg/mL) (MTT); significant caspase-3 activity	[191]
*Solanum lyratum* Thunb.	whole plant	chloroform	-	HSC-3, SAS, CAL-27	Cytotoxic (IC_50_ values in the range of 40–80 μg/mL); induction of apoptosis (in extrinsic- and intrinsic-dependent pathways) by changing levels of the proteins p21, p16, CDK2 and CDK6, and cyclins D1 and E. It also promotes proapoptotic proteins Bax and Bad and inhibits anti-apoptotic proteins Bcl-2 and Bcl-xl, promotes ROS and Ca2þ production, decreases mitochondrial membrane potential, increases NO production	[192]
*Solanum nigrum* L.	whole plant	water	alkaloids, glycosides, flavonoids, polyphenols terpenoids, and saponins	A-375	Cytotoxic	[193]
*Solanum nigrum* L.	whole plant	water	-	MCF-7	Cytotoxic (IC_50_ = 100 μg/mL) (crystal violet staining assay) induction of apoptosis by activation of caspase-3 and loss of mitochondrial integrity. It also inhibited EMT (cancer cell metastasis and migration) by downregulating ZEB1, N-cadherin, and vimentin	[194]
*Solanum nigrum* L.	leaves	water	-	SCC-4	Cytotoxic (IC_50_ = 150 μg/mL) (crystal violet staining assay); induction of apoptosis by increasing ROS production, activating caspase-9 and caspase-3, alleviating the inhibition of glucose uptake and loss of mitochondrial integrity	[195]
*Solanum nigrum* L.	fruit	ethanol	phenolic and flavonoid compounds	MCF-7	Cytotoxic (IC_50_ value = 40.77 μg/mL) (MTT assay); arrest the cell cycle in the S phase and continued to the G2/M phase	[196]
*Solanum nigrum* L.	whole plants	water	-	HepG2	Cytotoxic (MTT test); inhibits the proliferation and AKT/mTOR pathway	[197]
*Solanum paniculatum* L.	fruit	ethanol	carotenoids, phenolic compounds	MCF-7	Cytotoxic (IC_50_ value = 1.87–30 μg/mL)	[198]
*Solanum schimperianum* Hochst. ex A.Rich *Solanum villosum* Mill. *Solanum coagulans* Forssk. *Solanum glabratum* Dunal., *Solanum incanum* L., *Solanum nigrum* L.	aerial parts	ethanol	rutin	HepG2, HEK293, MCF-7	Cytotoxic (IC_50_ values in the range of 20.4–30.1 μg/mL) (MTT assay)	[199]
*Solanum sessiliflorum* Dunal	pulp/seed	ethanol	caffeic and gallic acids, beta-carotene, catechin, quercetin, and rutin	MCF-7, HT-29	Cytotoxic (IC_50_ values in the range of 3–>30 μg/mL) (MTT assay)	[200]
*Solanum tuberosum* L.	tuber, peels, flesh, flowers	water	-	HT-29	Cytotoxic (IC_50_ values in the range of 7.2–14.4 mg/ mL) (MTS test); induction of apoptosis by upregulation of caspase-3 protease activity	[201]
*Solanum tuberosum* L.	tubers	water	polyphenol and anthocyanin-rich	U937	Cytotoxic; expression of specific apoptotic agents, such as caspase 8, 9, 3, and poly (ADP-ribose) polymerase (PARP)	[202]
*Solanum tuberosum* L.	peels	ethanol/water	caffeic, caffeoylquinic acid, O-glycosylated flavonol derivatives and polyamine derivatives	NCI-H460, MCF-7, HepG2, and HeLa	Cytotoxic (GI_50_ values in the range of 51–365 μg/mL)	[203]
*Withania coagulans* (Stocks) Dunal	roots, leaves, leaf stalk, and fruit	methanol	flavonoid and phenolic content, myricetin, quercetin, gallic acid, hydroxybenzoic acid	HeLa, MCF-7, RD, RG2	Cytotoxic (IC_50_ values in the range of 0.96 μg/mL–6.69 μg/mL (Presto Blue cell metabolic test)	[204]
*Withania coagulans* (Stocks) Dunal	fruits	methanol	withaferin A	MDA-MB-231	Cytotoxic (IC_50_ = 40 mg/mL) (MTT assay)	[205]
*Withania somnifera* (L.) Dunal	root	water	-	A375	Cytotoxic (IC_50_ = 350 μg/mL) (MTT test); induction of morphological changes (apoptotic body and nuclear blebbing) and DNA fragmentation	[206]
*Withania somnifera* (L.) Dunal	leaves	water	-	HepG2	Cytotoxic (IC_50_ = 5.0 mg/mL)(MTT test); induction of apoptosis by caspase-3, -8 and -9 activation	[207]
*Withania somnifera* (L.) Dunal	roots and leaves	water, ethanol, metanol (various methods of extraction and maceration)	withanoside V, withanoside IV, 12-deoxywithastramonolide, withanolide A, and withaferin A	HeLa	Cytotoxic (IC_50_ = 10 mg/mL) (MTT test)	[208]
*Withania somnifera* (L.) Dunal	roots	ethanol/water	alkaloids, carbohydrates, phytosterols and phenolics	A549	Cytotoxic (IC_50_ = 99.7 μg/mL) (MTT test); anticancer activity via antioxidant, apoptotic, autophagy and angiogenesis inhibition mechanisms	[209]
*Withania somnifera* (L.) Dunal	roots	-	withaferin A, whitanolide, withanolide B	Jurkat	Proapoptotic mechanism involves intracellular Ca^2+^ accumulation and the generation of reactive oxygen species	[210]
*Withania somnifera* (L.) Dunal	leaves	water	-	C6 glioma	Activation of multiple pro-apoptotic pathways, leading to suppression of cyclin D1, Bcl-xl, and p-Akt	[211]
*Withania somnifera* (L.) Dunal	stems	methanol, ethanol, water	withaferin A	MDA-MB-231	Cytotoxic (IC_50_ values of 30 and 37 μg/mL) (MTT test)	[212]

**Table 2:** In vitro anticancer effect of pure compounds isolated from the Solanaceae family, and their potential mechanisms of action.

Name of the Species	Part of the Plant	Compounds/Fraction	Cancer Cell Lines	Activity/Mechanism/Effect	Ref.
*Brugmansia suaveolens* (Humb. & Bonpl. ex Willd.) Bercht. & J.Presl	leaves	SUPH036-022A	MCF7, A549	Cytotoxic (MTT test) and induction of apoptosis by loss of mitochondrial integrity and increase of ROS	[229]
*Capsicum annuum* L.	pericarp	polyphenolic content	U937	Cytotoxic (Trypan blue assay)	[230]
*Capsicum chinenses* L.	fruits	capsaicin and dihydrocapsaicin	SH-SY5Y	Cytotoxic (IC_50_ = 69.75 μg/mL) (Trypan blue assay)	[231]
*Datura innoxia* Mill.	aerial parts	dinnoxolide A, 21,27-dihydroxy-1-oxowitha-2,5,24-trienolide, daturamalakin B, withametelin	U251 and SK-LU-1	Cytotoxic (IC_50_ values in the range of 1.2–19.6 μM) (SRB assay)	[232]
*Datura inoxia* Mill.	leaves	phytosterol, rinoxiaB	HCT 15	Cytotoxic (IC_50_ = 4 μM), apoptotic effects by targeting BAX/Bcl2 pathway	[233]
*Datura metel* L.	seeds	indole alkaloids, daturametelindoles A–D	SGC-7901, Hepg2, MCF-7	Cytotoxic (IC_50_ values in the range of 6.73–47.63 μM/mL) (MTT test)	[234]
*Datura metel* L.	whole plants	steroidal saponins (metelosides A–E)	HepG2, MCF-7, and SK-Mel-2	Cytotoxic (SRB assay)	[235]
*Lycium ruthenicum* Murray	fruits	petunidin 3-*O*-[6-*O*-(4-*O*-(trans-p-coumaroyl)-α-l-rhamnopyranosyl)-β-d-glucopyranoside]-5-*O*-[β-d-glucopyranoside]	DU-145	Cytotoxic (IC_50_ = 361.58 μg/mL) (MTT test), apoptosis through the ROS/PTEN/PI3K/Akt/caspase 3 signalling pathway	[236]
*Lycium shawii* Roem. & Schult	whole plant	aloe emodin, dehydrocostus lactone costunolide, lyciumate, aloe emodine 11-*O*-rhamnoside, emodin-8-*O*-β-d-glucoside and lyciuma	MDA-MB-231	Cytotoxic (IC_50_ values in the range of >72 μg/mL) (MTT test)	[237]
*Physalis alkekengi var. franchetii* Mast.	aerial parts	physalin A	A549	Cytotoxic (IC_50_ = 28.4 μM/mL) (MTT test); cell cycle arrest in the G2/M phase and increase of ROS	[238]
*Physalis alkekengi var. franchetii* Mast.	-	physakengose G	U-2OS, HOS	Cytotoxic (MTT test), increase of lysosome dysfunction, induction of apoptosis (mitochondrial-dependent pathway) and inhibition of mTOR signalling	[239]
*Physalis alkekengi var. franchetii* Mast.	calyx	withanolides	A549, K562	Cytotoxic (IC_50_ value in the range of 1.9–4.3 μM/mL) (MTT test); induction of apoptosis by suppressing the PI3K/Akt/mTOR signalling pathway	[240]
*Physalis angulata* L.	stems and leaves	physangulatins A−N; withaphysalin Y;withaphysalin Z	C4-2B, 22Rvl, 786-O, A-498, ACHN, A375-S2	Cytotoxic (IC_50_ values in the range of 0.18–11.59 μM/mL) (MTT test)	[241]
*Physalis angulata* L.	stems and leaves	physalins and analogues (physalins V-IX, 16,24-cyclo-13, 14-seco withanolides)	C4-2B, 22Rv1, 786-O, A-498, ACHN, A375-S2	Cytotoxic (IC_50_ values in the range of 0.24–3.17 μM/mL) (MTT test)	[242]
*Physalis angulata* L.	whole plant	physalin B, physalin F	HL60, A549, HeLa, HuCCA-1, HepG2, MDA-MB-231), T47-D), S102, H69AR, MRC-5	Cytotoxic (IC_50_ values in the range of 0.76–11.92 μM/mL) (MTT, XTT test)	[243]
*Physalis angulata* L.	aerial parts	withanolide	MG-63, HepG-2, MDAMB-231	Cytotoxic (IC_50_ values in the range of 3.50–15.74 μM/mL)	[244]
*Physalis angulata* L.	whole plant	withanolides	A549, HeLa and p388	Cytotoxic (IC_50_ values in the range of 1.91–>30 μM/mL) (MTT test); apoptosis-inducing activity by flow cytometric analysis	[245]
*Physalis crassifolia* Benth.	fruits	17β-Hydroxy-18-acetoxywithanolides	LNCaP, PC-3M, MCF-7, NCI-H460 and SF-268	Cytotoxic (IC_50_ values in the range of 0.12–>5.0 μM/mL) (AlamarBlue)	[246]
*Physalis ixocarpa* Lam.	fruits	ixocarpalactone A	SW1990, MCF-7, HeLa	Cytotoxic (IC_50_ values in the range of 3.22–7.51 μM/mL) (CCK-8 assay); induction of apoptosis by inhibition of PHGDH	[247]
*Physalis minima* L.	whole plant	withanolides	A549, SMMC-7721, MCF-7	Cytotoxic (IC_50_ value in the range of 40.01–82.17 μM/mL) (MTT test)	[248]
*Physalis minima* L.	whole plant	5, 6-β-epoxywithanolides	A549, SMMC-7721, MCF-7	Cytotoxic (IC_50_ values in the range of 31.25–80.14 μM/mL) (MTT test)	[249]
*Physalis minima* L.	aerial parts	withanolide E, withaperuvin C, 4b-hydroxywithanolide E, 28-hydroxywithaperuvin C, physaperuvin G, and 4-deoxywithaperuvin	HepG2, SK-LU-1, and MCF7	Cytotoxic (IC_50_ in the range of 0.051–0.86 μg/mL)	[250]
*Physalis minima* L.	aerial parts	physaminilides HeK, withanolides	A375	Cytotoxic (IC_50_ values in the range of 1.2–7.5 μM/mL) (MTT assay)	[251]
*Physalis peruviana* L.	seeds	perulactones I–L, 17-deoxy-23β-hydroxywithanolide E, 23βhydroxywithanolide E, 4-deoxyphyperunolide A, 7β-hydroxywithanolide F, 7βhydroxy-17-epi-withanolide K, 24,25-dihydro-23β,28-dihydroxywithanolide G, and 24,25-dihydrowithanolide E, withanolides	LNCaP, 22Rv1 ACHN, M14, SK-MEL-28	Cytotoxic (IC_50_ values in the range of 0.11–> 2 μM/mL) (MTS assay)	[252]
*Physalis peruviana* L.	aerial parts	4-hydroxywithanolide E	HT-29, HCT116, Caco-2	Cytotoxic (IC_50_ = 0.84 μM/mL) (CCK-8); cell cycle arrest in the G0/G1 phase (at low concentrations) and induction of apoptosis (at higher concentrations) by changes in apoptosis-related proteins and genes and histone modification	[253]
*Physalis philadelphica* Lam.	aerial parts	7-epi-philadelphicalactone A; withaphysacarpin philadelphicalactone C, ixocarpalactone A	LNCaP, ACHN, UO-31, M14,SK-MEL-28	Cytotoxic (IC_50_ values in the range of 0.06–>10 uM/mL) (MTS assay)	[254]
*Physalis pubescens* L.	Fruits	physapubescin B	SKOV3, HepG2, MDA-MB-231, PC-3, Du145	Cytotoxic (IC_50_ values in the range of 1.85–16.05 μM) (MTT test); cell cycle arrest in the G2/M phase (associated with reduced Cdc25C levels and increased levels of CyclinB1, p21 as well as p-Cdk1)	[255]
*Physalis pubescens* L.	stems and leaves	physapubescin E physapubside A physapubside B physapubescin F physapubside C physapubescin G physapubescin H physapubescin I and two withanolides	C4-2B, 22Rvl, 786-O, A-498, ACHN, Caki-2, A375-S2, A375	Cytotoxic (IC_50_ values in the range of 0.17–5.30 μM/mL) (MTT test)	[256]
*Physalis pubescens* L.	fruits	physapubescin B	ES-2, A2780, A2780/TR	Induction of apoptosis and cell-cycle arrest	[257]
*Physalis pubescens* L.	fruits	physapubescin I	SW1990	Cytotoxic (IC_50_ in the range of 2.06–5.04 μM/mL)	[258]
*Salpichroa scandens* Dammer	aerial parts	salpichrolides A, C, D, G, M, S, T, and 2,3-dihydrosalpichrolide B and derivatives	LNCaP, PC-3, MCF-7, T47D	Cytotoxicity (IC_50_ values in the range of 29.97–64.91 μM/mL) (MTS assay)	[259]
*Solanum capsicoides* All.	seeds	carpesterol	U251, MCF-7, 786-0, OVCAR 03, K562	Cytotoxic (GI_50_ values in the range of 24.0–226.3 μg/mL)	[184]
*Solanum incanum* L., *Solanum schimperianum* Hochst, *Solanum nigrum* L., *Physalis lagascae* Roem. & Schult. and *Withania somnifera* (L) Dunal	leaves	steroidal glycoalkaloid fractions	MCF-7,MDA-MB-231, HT-29, HTC-116	Cytotoxic (IC_50_ values in the range of 1.29–>50 μg/mL) (MTT test)	[188]
*Solanum lycopersicum* L.	different parts	α-tomatine	CT-26	Inhibition of tumour growth and induction of apoptosis through caspase-independent signalling pathways	[260]
*Solanum lyratum* Thunb	whole plant	sesquiterpenoids including solajiangxin H and lyratol D	MCF-7, HCT-8, A549, SGC-7901, BEL-7402)	Cytotoxicity (IC_50_ value in the range of 4.8–5.9 μg/mL) (CCK-8); induction of apoptosis (mitochondrial-dependent pathway) by changes in apoptosis-related proteins	[261]
*Solanum lyratum* Thunb.	whole plant	steroidal compounds	SGC-7901, BEL-7402	Cytotoxic (IC_50_ value in the range of 0.39–71.89 μmol/mL) (MTT test)	[262]
*Solanum melongena* L.	fruit peels	solasonine; solasodine; solamargine	Huh7, HepG2	Cytotoxic (IC_50_ values in the range of 9.6–91.8 μM/mL) (SRB assay); cell cycle arrest in S-phase, induction of apoptosis,	[263]
*Solanum melongena* L.	sepals	melongenamides H-I	HeLa, Ishikawa and MGC-803	Cytotoxic (IC_50_ values in the range of 15.3–32.1 μM/mL) (CCK8 assay)	[264]
*Solanum nigrum* L.	whole plant	degalactotigonin, solasodine, O-acetyl solasodine, and soladulcoside A	PANC1, MIA-PaCa2, A549, NCI-H1975, and NCI-H1299	Cytotoxic (IC_50_ values in the range of 2.9–>30) (Cell Migration Assay),; induces apoptosis and cell cycle arrest via inhibiting the EGFR signalling pathways	[265]
*Solanum nigrum* L.	fruits	solaoiacid	A549	Cytotoxic (IC_50_ = 2.3 μmol/mL (MTT assay)	[266]
*Solanum nigrum* L.	fruits	alkaloid glycosides	HL-60, U-937, Jurkat, K562, and HepG2	Cytotoxic (IC_50_ values in the range of 2.72–39.19 μM/mL) (MTT assay)	[267]
*Solanum nigrum* L.	leaves	uttroside B	HepG2	Cytotoxic (IC_50_ = 0.5 μM) (MTT test); induction of apoptosis by down-regulating the activation of MAPK and mTOR pathways	[268]
*Solanum nigrum* L.	-	degalactotigonin	different lines of osteosarcoma cells	Cytotoxic (IC_50_ values in the range of 12.91–31.46 μM/mL) (MTT test); induction of apoptosis, suppression of migration and invasion by repression of the Hedgehog/Gli1 pathway through GSK3b inactivation.	[269]
*Solanum nigrum* L.	fruits	solanine A; 7a-OH khasianine, 7a-OH solamargine; 7a-OH solasonine	MGC803, HepG2, SW480	Cytotoxic (IC_50_ values in the range of 6.00–9.25 μM/mL) (SRB assay)	[270]
*Solanum septemlobum* Bunge	whole plant	septemlobin D and 11,12-O-isopropylidenesolajiangxin F	P-388, HONE-1 and HT-29	Cytotoxic (IC_50_ values in the range of 3.0–7.3 μM/mL) (MTT test)	[271]
*Solanum torvum* Swartz.	Fruits	methyl caffeate	MCF-7	Cytotoxic (IC_50_ = 0.62 μM/mL) (MTT test); induction of apoptosis by caspase activation via cytochrome c release from mitochondria. Further, increased DNA fragmentation, apoptotic body and changes in apoptosis-related proteins (Bcl-2, Bid and Bax)	[272]
*Withania adpressa* Coss.	leaves	glycowithanolide named wadpressine, withanolide F, withaferin A, coagulin L and nicotiflorin	MM-CSCs, RPMI 8226	Cytotoxic (IC_50_ values in the range of 0.1–>20 μM/mL) (MTT test)	[273]
*Withania somnifera* (L.) Dunal	roots	withasilolides A−F, withanone	A549, SK-OV-3, SK-MEL-2, and HCT-15	Cytotoxic (IC_50_ values in the range of <10.0 μM/mL) (SRB assay)	[274]
*Withania somnifera* (L.) Dunal	leaves	withaferin A and its derivatives	PANC-1, DU145, MCF7	Cytotoxic (IC_50_ values in the range of 1.1–>25 μM/mL)	[275]
*Withania somnifera* (L.) Dunal	roots	protein fraction	MBA-MB-435, MDA-MB-231, T47D, MCF-7, HCT-116, A549	Cytotoxic (IC_50_ = 92 μg/mL) (MTT test); induction of apoptosis by decrease of the mitochondrial membrane potential levels, promotion of the reactive oxygen species production, changes in apoptosis-related proteins regulation and caspases-3 activation. Further, cell cycle arrest in G2/M-phase.	[276]
*Withania somnifera* (L.) Dunal	roots and leaves	withanoside V, withanoside IV, 12-deoxywithastramonolide, withanolide A, and withaferin A	HeLa	Cytotoxic (IC_50_ value in the range of 3.2 to 7.7 μM/mL) (MTT test)	[208]

**Table 3:** Anticancer effect of nanoparticles in combination with plant extracts from the Solanaceae family, and their potential mechanisms of action.

Name of the Species	Part of the Plant	Type of Solvent/Active Compounds	Type of Nanoparticles	Cancer Cell Lines	Activity/Mechanism/Effect	Ref.
*Atropa acuminate* Royle ex Lindl.	leaves	water/total phenolic, flavonoid and tannin	Ag	HeLa	Cytotoxic (IC_50_ = 5.418 μg/mL) (MTT test)	[279]
*Lycium chinense* Mill.	fruits	water	Au, Ag	MCF 7	Cytotoxic (MTT test)	[280]
*Lycopersicon esculentum* L.	fruits	benzene/lycopene	Ag, Au, Fe	COLO320DM, HT29 and HeLa	Cytotoxic (MTT test)	[281]
*Solanum elaeagnifolium* Cav.	leaves	water	Ag-AgO-Ag_2_O	A-549	Cytotoxic (IC_50_ = 67.09 μg/mL) (MTT test)	[282]
*Solanum incanum* L.	leaves	water	Ag-NPs	HepG2, MCF-7	Cytotoxic (IC_50_ values in the range of 21.76–129.9 μg/mL) (MTT test)	[283]
*Solanum lycocarpum* A.St.-Hil.	fruits	glycoalkaloids	NP-AE	RT4	Cytotoxic (2D model: IC_50_ = 4.18 μg/mL, 3D model: three-fold higher than in 2D cell culture) (2D—the neutral red assay, 3D—CellTiter-Glo^®^3D); induction of apoptosis by cell cycle arrest	[284]
*Solanum lycocarpum* L.	fruits	ethanol-soluble fraction glycoalkaloids, solamargine and solasonine	AE-loaded folate-targeted nanoparticles	MDA-MB-231, RT4	Folate-conjugated polymeric nanoparticles are potential carriers for targeted glycoalkaloidic extract delivery to bladder cancer cells (2D model: IC_50_ = 3.78 μg/mL, 3D model: 7.7 μg/mL) (2D model—Neutral Red Uptake assay, 3D model: CellTiter-Glo^®^3D)	[285]
*Solanum lycopersicum* L.	tomato’s pomase	ethyl acetate/lycopene	lycopene-NPs	MCF-7, HCT-116, HepG2,	Cytotoxic (IC_50_ in the range of 72.40–92.54 μg/mL) (MTT test)	[286]
*Solanum muricatum* L.	leaves	water	Ag	HeLa	Cytotoxic (IC_50_ = 37.5 μg/mL) (MTT assay)	[287]
*Solanum trilobatum* L.	unripe fruits	water	Ag	MCF7	Cytotoxic (MTT test); induction of apoptosis by changes in expression of proliferation- and apoptosis-related genes (overexpression Bax¸ down-regulation Bcl-2), and activation of caspases 3 and 9	[288]
*Solanum trilobatum* L.	leaves	water	Mn-Ag co-doped FeO	MCF-7, HeLa	Cytotoxic (IC_50_ value in the range of 37.11–60.49 μg/mL) (MTT test)	[289]
*Solanum xanthocarpum* Schrad. & Wendl	leaves	water	Au	C666-1	Cytotoxic (MTT test); triggering cell death by autophagy and apoptosis (mitochondrial-dependent pathway)	[290]
*Withania somnifera* L.	leaves	water/total flavonoid, phenolic and tannin	Se	A549	Cytotoxic (IC_50_ = 25 μg/mL) (MTT test)	[291]
*Withania somnifera* L.	leaves	water/phenolic, flavonoid and tannin	Zn	HEP2, PC3, MCF-7, HCT-116,	Cytotoxic (IC_50_ value in the range of 19.17–88.3 7 μg/mL) (MTT test)	[292]

**Table 4:** Anticancer synergistic effect of chemotherapeutic drugs and plant extracts from the Solanaceae family.

Name of the Species	Part of the Plant	Type of Slovent or Fraction or Compound	Chemotherapeutic Drugs	Cancer Cell Lines	Activity/Mechanism/Effect	Ref.
*Capsicum frutescens* L.	-	capsaicin	doxorubicin	Caco-2 and CEM/ADR 5000	Enhancement of the doxorubicin cytotoxicity in cancer cells and chemosensitizing activity (inhibition of P-glycoprotein activity)	[297]
*Lycium barbarum* L.	fruits	water	doxorubicin	MCF-7, MDA-MB-231	Enhancement of the doxorubicin cytotoxicity in cancer cells	[298]
*Solanum cernuum* Vell.	leaves	cernumidine	cisplatin	T24, RT4, 5637	Enhancement of the cisplatin cytotoxicity in cancer cells. Inhibition of cell migration, down-regulation of MMP-2/9 and p-ERK1/2, increase EGFR activity. Furthermore, down-regulation of Bcl-2, up-regulation of Bax and reduction of the mitochondrial membrane potential	[299]
*Solanum incanum* L.	extract—according to the patent (US patent 7,078,063, EU patent 1,058,334, and Japan patent 3,940,928) SR-T100	extract containing solamargine	cisplatin, paclitaxel	ES2, TOV-21G, IGROV1, A2780, A2780CP70, ov2008 and ov2008CP20	Suppression of C/EBPβ and COL11A1 expression and its promoter activity	[300]
*Solanum nigrum* L.	leaves	water	cisplatin, doxorubicin	Hep3B, HepJ5	Induction of caspase-7 and accumulation of microtubule associated protein-1 light chain-3 A/1B II	[301]
*Solanum nigrum* L.	leaves	water	cisplatin, doxorubicin, docetaxel	ES-2, SKOV-3, OVCAR-3	Induction of caspase-3 and accumulation of microtubule associated protein-1 light chain-3 A/1B II	[302]
*Solanum nigrum* L.	unripe fruit	glycoside fraction (methanol)	doxorubicin	NCI/ADR-RES	Overcoming doxorubicin resistance by inhibiting the JAK-STAT3 signalling pathway by downregulation of JAK1, STAT3, pSTAT3, and Mdr1 expression. Furthermore, the cell growth suppression was proven to be apoptotic, based on results obtained from DNA fragmentation, annexin V apoptosis assay and PARP cleavage analysis.”	[303]

**Table 5:** In vivo anticancer effect of plant extracts and pure compounds from the Solanaceae family and their potential mechanisms of action.

Name of The Species	Part of the Plant	Type of Solvent	Class of Compounds/Compounds Identified in Extract/Fraction	Potential Mechanism of Action	Ref.
*Athenaea velutina* Sendtn.	leaves	dichloromethane: methanol (1:1)	phenolic compounds and flavonoids	Suppression of the development of pulmonary melanomas following the intravenous injection of melanoma cells to C57BL/6 mice	[161]
*Datura stramonium* L., *Datura inoxia* Mill.	leaves	ethyl acetate	rutin, gallic acid, catechin, apigenin and caffeic acid	Alleviative effects in benzene induced leukaemia in Sprague Dawley rats	[166]
*Physalis alkekengi* L.	aerial parts	hydro alcoholic	-	Tumour progression on the 28 ER+ BC BALB/c mice animal model (the tumour size among the different doses of extract lose to 0.6 mm was in the greatest dimension with dosage of 10 mg/kg)	[310]
*Physalis ixocarpa* Lam.	fruits	-	ixocarpalactone A	Inhibition of the tumour growth in a SW1990 xenograft mouse model with low toxicities, suggesting its potential therapeutic application in pancreatic cancer treatment	[247]
*Physalis pubescens* L.	fruits	-	physapubescin B	Antitumour efficacy in human prostate cancer PC3 xenograft in nude mice	[255]
*Solanum incanum* L.	whole plant SR-T100	acid base precipitation followed by the different ratios of ethanol/H_2_ O extraction (according to the patents—US patent 7,078,063, EU patent 1,058,334, and Japan patent 3,940,928)	solamargine	Extract SR-T100-treated C57BL/6 mice, the tumour burden of lung metastases was significantly reduced compared to that in control mice	[186]
*Solanum incanum* L.	whole plant SR-T100	acid base precipitation followed by the different ratios of ethanol/H_2_ O extraction (according to the patents—US patent 7,078,063, EU patent 1,058,334, and Japan patent 3,940,928)	solamargine	Animal experiments showed that all papillomas (35/35) and 27 of 30 UVB-induced microinvasive SCCs in hairless SKH-hr1 female mouse mice disappeared within 10 weeks after once-daily application of topical SR-T100 extract	[304]
*Solanum lycopersicum* L.	different parts	-	α-tomatine	Intraperitoneally administered α-tomatine (5 mg/kg body weight) also markedly inhibited growth of the tumour using CT-26 cancer cells without causing body and organ weight changes. The reduced tumour growth in the BALB/c mice by 38% after 2 weeks was the result of increased caspase-independent apoptosis associated with increased nuclear translocation of AIF and decreased surviving expression in tumour tissues.	[260]
*Solanum nigrum L.*	fruits	methanol	rutin, solasonine, quercetin and solamargine	Reduction of the growth and infiltration of C6 glioma tissue and suppressed the proliferation of tumour cells in Wistar rats brain	[311]
*Solanum nigrum* L.	stems	-	polysaccharide fraction (SN-ppF3)	Tumour suppression mechanisms observed in SN-ppF3-treated mice were most probably due through enhancing the host immune response	[312]
*Solanum nigrum* L.	leaves	-	uttroside B	Drastic inhibition of tumour growth produced by uttroside B in NOD-SCID mice bearing human liver cancer xenografts demonstrates the chemotherapeutic efficacy of uttroside B	[268]
*Solanum nigrum* L.	-	-	degalactotigonin	Degalactotigonin injected intraperitoneally after tumour inoculation, significantly decreased the volume of osteosarcoma xenografts in athymic nude (nu/nu) mice model and dramatically diminished the occurrence of osteosarcoma xenograft metastasis to the lungs	[269]
*Withania somnifera* L.	leaves	water	ASH-WEX extract	Reduced the intracranial tumour volumes in vivo and suppressed the tumour-promoting proteins p-nuclear factor kappa B (NF-κB), p-Akt, vascular endothelial growth factor in the albino rat model of orthotopic glioma allograft	[211]

## Data Availability

Not applicable.
